# Hepatitis B virus (HBV) infection amongst children in Senegal: current prevalence and seroprotection level

**DOI:** 10.11604/pamj.2019.32.140.14485

**Published:** 2019-03-25

**Authors:** Gora Lô, Amina Sow-Sall, Halimatou Diop-Ndiaye, Ndiaye Babacar, Niokhar Ndane Diouf, Sokhna Moumi Daffé, Babacar Ndao, Moussa Thiam, Moustapha Mbow, Mamadou Bayo Soumboundou, Maud Lemoine, Maguette Sylla-Niang, Ousseynou Ndiaye, Cheikh Saad Boye, Souleymane Mboup, Ndeye Coumba Touré-Kane

**Affiliations:** 1 Institut de Recherche en Santé, de Surveillance Epidémiologique et de Formation (IRESSEF), Dakar, Sénégal; 2 Centre Médical Inter Armées Lemonier, Dakar, Senegal; 3 Laboratoire de Bactériologie, Virologie de l’Hôpital Aristide Le Dantec, Dakar, Sénégal; 4 Hôpital Général de Grand, Yoff, Senegal; 5 Hôpital pour Enfant de Diamniadio, Dakar,Sénégal; 6 Imperial College London, St Mary’s Hospital campus, London, UK; 7 Hôpital Albert Royer de Dakar, Sénégal; 8 Hôpital Dalal Diamm, Dakar, Sénégal

**Keywords:** Vaccination, hepatitis B, efficacy, immunity, Senegal, children

## Abstract

**Introduction:**

Hepatitis B virus (HBV) infection is highly endemic in Senegal. HBV vaccine of all children has been introduced in 1999 and included in the Expanded Programme on Immunization in 2004. The aim of this study was to assess the HBV prevalence and immunity status against HBV amongst children in Senegal.

**Methods:**

Between March and August 2016, consecutive children aged from 6 months to 16 years old were recruited in outpatient department of three main children hospitals in Senegal. Serum samples were analyzed for HBV serology (HBsAg, HBcAb, HBsAb) using ARCHITECT analyzer. Children with HBsAb levels ≥ 10 IU/l) were considered as seroprotected against HBV.

**Results:**

During the study period, 295 children fulfilled the criteria for the study and were further analyzed. Three children were HBsAg positive giving a seroprevalence at 1.1% (95% CI: 0.2-3.3), 12/267 (4.5%, 95% CI=2.3-7.7) had positive HBcAb and 226/295 (76.6%, 71.4-81.3) had positive HBsAb including 191 (77.3%, 71.6-82.4) with isolated HBsAb related to previous active immunization. However only 165 children (56%, CI 50-62) had seroprotective HBsAb levels (HBsAb ≥ 10 UI/L) and 63 (21.4, 16.8-26) had a strong seroprotectiondefined by HBsAb ≥ 100 IU/L.

**Conclusion:**

Our results suggest that although HBV prevalence has significantly decreased in children in Senegal following a better HBV vaccine coverage, the number of children correctly seroprotected is insufficient (56%). Assessing the levels of HBsAb and providing HBV vaccine boosters should be considered in children in Senegal.

## Introduction

Hepatitis B virus (HBV) infection is a major public health problem and a leading cause of morbidity and mortality globally, affecting approximately 250 million persons worldwide [1] and accounting for 650,000 deaths annually [2]. Most of these deaths occur in resource-limited countries in Asia and Africa. Without effective preventive and therapeutic interventions chronic hepatitis B (CHB) infection will lead to an estimated 11.8 million deaths by 2030, primarily as a result of cirrhosis and hepatocellular carcinoma (HCC) [3]. The World Health Organization (WHO) has recently incorporated HBV elimination in its global health agenda and plans a 90% reduction of anew HBV cases and a 65% reduction of HBV-related mortality by 2030. In order to achieve these ambitious targets prevention of HBV transmission in particularly in endemic countries should be urgently improved. Effective HBV vaccines have been developed in the early 80s and in 2009, the WHO has recommended the introduction of the vaccine into the national immunization programs of all endemic countries. However, the current coverage of three doses of HBV vaccines remains imperfect with an estimate below 80% in 2015 in Africa [4]. In sub-Saharan Africa, the seroprevalence of Hepatitis B s antigen (HBsAg) in the adult population is high, estimated over 8% [1, 5]. In Senegal, 85% of the population has at least one marker of previous or current HBV infection and the prevalence of HBsAg varies between 11 to 17% [6].

Following three doses of vaccine given at 4 weeks interval, it has been demonstrated that 90-99% of healthy neonates, children, adolescents and adults will develop protective levels of HBs antibody (HBsAb) [7] defined by serum levels ≥ 10 IU/L although a level below 10 IU/L does not necessarily indicates loss of immunity [8]. In Senegal, one of the most endemic countries for HBV in the world, the HBV vaccine has been introduced in 1999 and included in the Expanded Programme on Immunization (EPI) only in 2004 with recommended injections in neonates at the age of 1, 2 and 3 months using a recombinant vaccine. Since January 2016 the Senegalese ministry of health has been providing the HBV birth dose vaccine within the 24 hours of life to all infants born in Senegal in order to comply with the WHO guidelines and eventually control the burden of HBV-related liver diseases in Senegal. Following the 2016 call from the WHO and world health assembly to control and eliminate HBV worldwide, the aim of the following study was to evaluate the current immunization profiles against HBV in a large number of Senegalese children aged from 6 months to 16 years.

## Methods

**Inclusion criteria:** Inclusion criteria of the study were: between 6 months and 15 years old, no risk of haemorrhage when taking samples (hemophilia). Consent was obtained from each each parent or accompanying childrenchild. Children took at least a dose of HBV vaccine.

**Type and study population:** This is a preliminary cross-sectional study realized between the 1^st^ of March 2016 and 30^th^ of August 2016 of the hepatitis B assessment study in Senegal. The target population was children aged 6 months to 16 years recruited in three main children hospitals in Dakar (Hôpital Général de Grand Yoff, Hôpital pour Enfant de Diamniadio and Clinique de l'Amitié). The size of the study population was estimated at 1/8 of that of the large study where 3200 children will must be recruited in 3 regions of Senegal: Dakar, Thiès and Kaolack. Consecutive children were recruited in outpatient departments; demographic data and blood were collected after obtaining parents' consents. All included children received a blood sample of 3 milliliters of blood by venipuncture in vacuum tubes. After centrifugation, the sera are stored locally at -20^o^C and subsequently sent and analyzed in the laboratory of Medical Center joint Army Lemonier.

**Ethical approval:** This study was approved by University Senegalese ethic committee. The reference of authorization was Protocol 0261/2017/CER/UCAD: evaluation of the vaccine response in children in Senegal.

**Serological assays:** Qualitative assays for HBV serology (HBsAg, HBsAb and HBcAb) and quantitative assays for HBsAb were performed using the ARCHITECT analyzer and its commercial kit (Abbott Diagnostics, Wiesbaden, Germany). The ARCHITECT analyzer provides HBsAb levels measured in international units per liter (IU/L) with a linear range from 2 to 1,000 IU/L.

**Definition of hepatitis B seroprotection:** Children with HBsAb levels ≥ 10 IU/L were considered as seroprotected against HBV and good level of seroprotection was defined as HBsAb level ≥ 100 UI/L [7]. Children with HBsAb level < 10 IU/L were considered as non-seroprotected. However, children with HBsAb levels between 2 and 10 IU/L were considered to be immunized with poor seroprotection level. Children with HBsAb < 2 IU/L were considered as non-immunized.

**Virological assays:** HBV DNA was measured in all HBsAg-positive samples and all samples negative for HBsAg but positive for either HBsAb and/or HBcAb in order to identify potential occult hepatitis B. DNA was extracted from 200 μL of serum samples using a DNA/RNA extractor kit (AMPLIX^®^ HBV Biosynex, Strasbourg France). The HBV DNA detection is based on the amplification of a specific conservative DNA sequence of an open reading frame X (ORFx) and by measuring the amplification product concentration in the course of the PCR process by means of fluorescence marked probe. Both assays have a very good limit of detection at 26 IU/L (AMPLIX^®^Hepatitis B Virus (HBV): (Real time PCR kit designed for the detection of the Hepatitis B virus (HBV) DNA. Reference 5625).

**Statistical analysis:** Statistical analysis of the results was performed using commonly used variation methods in Excel 2003 (Microsoft, USA) and Epi Info version 7. Continuous variables were described as medians (standard deviation, SD) while categorical variables were described as numbers (percentages). For group comparison, continuous variables were conducted by Mann-Whitney tests and categorical variables were conducted by Chi-squared test distribution with Yates correction (significance threshold p < 0.05).

## Results

**Characteristics of the study population:** Between March and August 2016, out of 416 consecutive children who visited the study centers, 295 were aged between 6 months and 16 years old and their parents accepted to participate to the study. General characteristics of the study children are summarized in Table 1. The vast majority of the children (34 (46%)) were aged below 5 years and 61 (21%) were more than 10 years.

**Table 1:** Characteristics of the study children

Parameters	Study children; n=295
**Mean age (years, SD)**	7.26 (5.07)
Age <5 years, n (%)	139 (47%)
Age 5-10 years, n (%)	95 (33%)
Age ≥ 10 years, n (%)	61 (21)
Male sex, n (%)	159 (54%)
**Markers of HBV infection**	
Positive HBsAg, n (%, CI), n=295	3 (1.1, 0.2-3.3)
Positive HBcAb, n (%, CI), n=267	12 (4.5, 2.3-7.7)
**Markers of seroprotection, n=295**	
Total Positive HBsAb, n (%, CI)	226 (76.6, 71.4 - 81.3)
Isolated positive HBsAb, n (%, CI)	191 (77.3, 71.6 – 82.4)
Median HBsAb levels (IU/L, IQR)	17 (0 – 1,000)
**Level of seroprotection agains HBV, n =295**	
Seroprotected children, n (%, CI) i.e positive HBsAb ≥ 10 IU/L	165 (56, 50-62)
Number of children with strong seroprotection i.e HBsAb≥100 IU/L	63 (21.4, 16.8 – 26)
Immunized but non-seroprotected children, n (%, CI) i.e positive HBsAb 2-10 IU/L	61 (20.7, 16.2 - 25.8)
Non-infected and non-immunized children, n (%, IC) i.e neg. HBsAg, neg HBcAb and neg. HBsAb	69 (23.4, 17.8 – 28.6)

### Markers of HBV infection

**Seroprevalence of HBs antigen (HBsAg):** Among 295 children analyzed, 3 had a positive HBsAg serology giving an overall prevalence of HBsAg at 1.1% (95% CI: 0.2-3.3) (Table 1). In children aged under 5 years, HBsAg prevalence was slightly higher at 1.6% (95% CI: 0.2-5.8). In children aged 5-10 years, 2 had a positive HBsAg giving a seroprevalence at 1.6% (95% CI: 0.2-5.8). No positive cases of HBsAg were noted in children over 10 years, but the sample size in that age group was small (n = 61). There was no significant difference in the prevalence of HBsAg by age and sex (1.4% versus 0.8%, p = 0.55).

**Seroprevalence of HBcAb (HBcAb):** Out of 295 children, HBcAb could be tested in 267 and 12 (4.5%, 95% CI = 2.3-7.7)) were positive for this marker (Table 1). The HBcAb seroprevalence was (n = 5, 4.1%, 95% CI = 1.3-9.3) in girls as compared to 8% (95% CI = 2.0-9.7) in boys; this difference was not statistically significant (p = 0.39). The lower rate of HBcAb (0.8%) (95% CI = 0.0-4.4) was noticed in the 0-5 years old group. This rate increased with age and reached 13.7% (95% CI = 5.7-26.3) in the age group 10 to 15 years. The difference between various age groups was statistically significant (p = 0.0004).

**HBV sero-protection:** Of the 295 children analyzed, 191 (77.3% and CI: 71.6-82.4) children had isolated HBsAb reflecting the proportion of children who were vaccinated at the time study.

**Figure 1:**
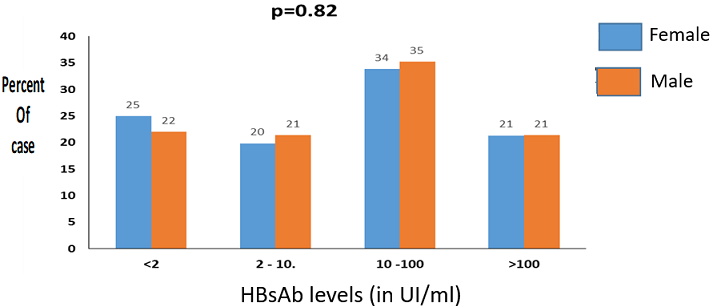
HBsAg seroprevalence by sex and age

**Figure 2:**
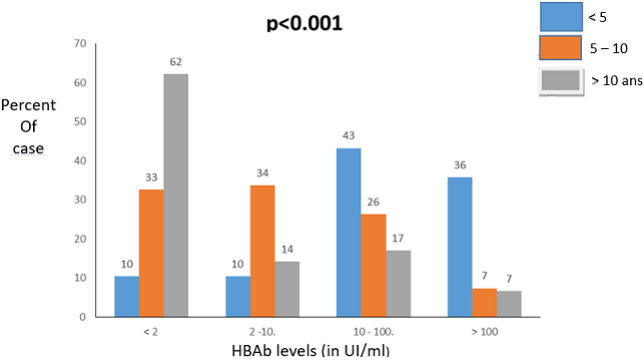
HBsAb levels by sex

**Level of HBs antibody:** The median of HBsAb levels in children was 17 IU/L (0-1,000 IU/L). Among the 295 study children, 165 (56% (95% CI = 50-62) were considered as seroprotected against HBV as defined by HBsAb levels ≥ 10 IU/L and 130 (44% (95% CI = 33-45)) had no or poor seroprotection i.e HBsAb level (< 10 IU/l). Of the 295 children, 69 (23% (95% CI = 19-29)) had HBsAb levels under 2 IU/L and were therefore considered as non-immunized. There was no statistical significant difference between both sexes (56.6% and 55.1% in boys and girls respectively, p = 0.82). Figure 1 and Figure 2 shows the level of HBsAb by sex and age. Of 165 children with HBsAb seroprotective levels (HBsAb-10 UI/L), the rate was much higher in children below 5 years (79.1%) as compared to children aged 5-10 years (33.7%) and children > 10 years (41%), p < 0.0001. HBV viral load was undetectable in all samples from 15 children tested either positive for HBsAg (n = 3) or children with positive HBcAb and negative HBsAg (n = 12).

## Discussion

This study aimed to assess the seroprevalence of HBsAg amongst children (6 months-16 years old) and the level of seroprotection against HBV in Senegal in 2016. Our findings provide two main messages 1) more than 10 years after the introduction of the vaccine within the EPI, the HBsAg seroprevalence amongst children in Senegal has significantly decreased (1.1%) as compared to the high prevalence reported in the 90s (> 15.%) [9]. This indicates a great impact of the integration of the HBV vaccine within the EPI as it has been observed in Asian and Western countries [10]. 2) Almost 80% of the children were vaccinated against HBV but only a small proportion (56%) were correctly sero-protected suggesting that many children in Senegal are either not correctly vaccinated or require additional HBV vaccine booster. In Senegal HBV infection is mainly acquired during early childhood and before the introduction of the HBV vaccine in the country (1999) HBsAg prevalence among children was estimated around 19% [11] and remained consistently high in older age groups. The control of hepatitis B infection has become one of the health priorities of the National Health Programme in Senegal. The programme currently includes universal immunization of infants, systematic HBsAg screening of blood product donors and care (including access to antiviral therapy) of people with chronic hepatitis B [6].

Vaccination is considered as the most cost-effective way to control HBV infection, and early immunization of newborns is critical to prevent perinatal HBV transmission. The successful introduction of HBV vaccine into the National Programme to fight against hepatitis B in Senegal had a great impact on the prevalence of HBsAg among population aged 1-15 years. Our results confirm that vaccination of infants contributes to the reduction of the HBsAg prevalence. Children hospitalized in two hospitals in Dakar tested for HBsAg between 2009-2010 had a low prevalence of HBsAg (0.2%) [12]. However, no significant difference was found between these 2 studies (p = 0.139). Compared with national studies, our overall HBsAg carrier rate was lower than those in 930 children born from HIV-seropositive mothers (3.0%) [13] and 462 babies including 88 born to 80 HBsAg (+) mothers (HBsAg (+) (17%) than to HBsAg (-) (4%) women [14]. Moreover compared with international studies, the HBsAg prevalence observed in our study (1.1%) was lower than those found in Nigerian children aged 2 months to 15 years (13.9%) [15], healthy Indonesian children (3.1%) [16] and HIV-infected children in Swaziland (1.4%) [17]. The discrepancy observed with other African countries is likely associated with several factors: a modest decrease in the HBsAg prevalence among African women, from 13.8% in 1992 to 11.57% in 2012 [14, 18] and different vaccine coverage over the African continent. According to the 2015 WHO-Unicef immunization report the HBV 3 doses vaccine coverage has improved in Senegal and was estimated at 89% in Senegal [6].

In our study we found that 77.3%, (95%CI 71.6-82.4) had isolated positive HBsAb suggesting a HBV vaccine coverage close to 80% in our study population. HBV vaccination or infection induces the production of HBsAb, an immunoglobulin able to neutralize HBsAg in case. Along with other effector immune functions, the protective HBsAb can block the spread of HBV in the body. HBsAb levels persist in the blood for a long time but gradually decrease with age. Our study confirms this effect (Figure 2). Our results are close to those reported by other authors in Senegal showing that the rate of positive HBsAb was 61 % in hospitalized children aged between 3 month to 6 years (p=0.302) [12]. Our results are however lower than those found in other HBV endemic countries where HBV vaccine coverage rates among children range from 90 to 98%. [19, 20]. We also found that the seroprotective rate of HBsAb levels was higher in males (56.6%) as compared to females (55.1%) (p > 0.05) suggesting gendre-guided immunity responses. In contrast previous studies reported better protective levels of HBsAb in vaccinated children (70%) [21] and 84.2% [22] with higher levels in girls as compared to boys. Such discrepancy could be attributed to differences in the primary immune response to vaccination, the type of vaccines used, the quality of vaccine storage (broken cold chain for instance) age groups, nutritional status and socioeconomic factors or race factors [23].

In our study protective level of HBsAb decreased with age from 65% in children below 5 years to 15.3% in those above 10 years. Similar findings were reported in Egypt and China where the protection rate was inversely correlated with age [24, 25]. A study conducted in Yemen assessed the hepatitis B vaccine coverage and immune response in children below ten years and reported a 45.2% children with low levels of HBs (< 10 UI/L) despite a full course of 3 doses vaccine [26]. These results suggested a poor response to HBV immunization in African children [27]. Interestingly we did not find any occult hepatitis B in the children enrolled in our study. In a recent study conducted in China, a low rate of occult hepatitis B (1.26%) was observed in 1.192 community children [28]. Our study has some limitations: first the parents were not surveyed on the history of hepatitis B in the family. Second we were not able to assess the number of HBV vaccine doses that were administrated to the children. It has been previously demonstrated that 3 doses are the best method to get effective protection against HBV and is better than one single dose. Third, we did not collect the HIV and nutritional/clinical status of the children and we were therefore unable to identify risk factors of poor HBV seroprotection in Senegalese children. Finally our study was conducted in urban centers which HBV epidemic and vaccine coverage certainly differ from Senegalese rural areas. This difference can be confirmed by the results of the large study.

## Conclusion

This study reports a low rate of HBV infection (1.1%) amongst children in Senegal ten years after the introduction of the HBV vaccine within the national immunization programme. Our findings suggest that the number of HBV vaccinated children (77%) remains insufficient and a large number of children have poor seroprotection against HBV. Additional efforts are needed to achieve 100% HBV vaccine coverage among the Senegalese infants in particular newborns. Whether the low HBsAb levels have a clinical impact in these children will have to be determined by re-assessing these children in the future. If this has an impact, assessing the levels of HBsAb and providing HBV vaccine boosters should be considered in children in Senegal.

### What is known about this topic

- The seroprotection rate of vaccinated subjects was less than 80%; hepatitis B is a real public health problem with prevalence above 8% in our countries; immunization coverage remains inadequate with seroprotection rates below 80%;
- Eighty percent of those vaccinated with HBV did not have seroprotective antibody levels;
- Only 90% of healthy people will develop protective levels of anti-HBs antibodies (≥ 10 IU/L) after taking 3 doses of HBV vaccines at 4 week intervals.

### What this study adds

- This study shows a very low prevalence of this infection in children;
- This study shows the need, on the one hand, to evaluate the seroprotection of subjects after taking the three doses in order to propose boosting immunity and on the other hand to make an evaluation of the quality of vaccines, the vaccine storage system and the their usage.

## Competing interests

The authors declare no competing interests.
